# Therapeutic potential of human umbilical cord mesenchymal stem cells on aortic atherosclerotic plaque in a high-fat diet rabbit model

**DOI:** 10.1186/s13287-021-02490-8

**Published:** 2021-07-15

**Authors:** Yanhong Li, Guiying Shi, Yunlin Han, Haiquan Shang, Huiwu Li, Wei Liang, Wenjie Zhao, Lin Bai, Chuan Qin

**Affiliations:** Key Laboratory of Human Diseases Comparative Medicine, Ministry of Health; Institute of Medical Laboratory Animal Science, CAMS&PUMC; Key Laboratory of Human Diseases Animal Models, State Administration of Traditional Chinese Medicine, Beijing Key Laboratory for Animal Models of Emerging and Remerging Infectious Diseases, Beijing, 100021 China

**Keywords:** Atherosclerosis, Human umbilical cords mesenchymal stem cells, Rabbit, Inflammation, TMAO

## Abstract

**Background:**

Atherosclerosis (AS) is a complex disease caused in part by dyslipidemia and chronic inflammation. AS is associated with serious cardiovascular disease and remains the leading cause of mortality worldwide. Mesenchymal stem cells (MSCs) have evolved as an attractive therapeutic agent in various diseases including AS. Human umbilical cord MSCs (UCSCs) have been used in cell therapy trials due to their ability to differentiate and proliferate. The present study aimed to investigate the effect of UCSCs treatment on atherosclerotic plaque formation and the progression of lesions in a high-fat diet rabbit model.

**Methods:**

Rabbits were fed a high-fat diet and then randomly divided into three groups: control, model, and treatment groups. Rabbits in the treatment group were injected with UCSCs (6 × 10^6^ in 500 μL phosphate buffered saline) after 1 month of high-fat diet, once every 2 weeks, for 3 months. The model group was given PBS only. We analyzed serum biomarkers, used ultrasound and histopathology to detect arterial plaques and laser Doppler imaging to measure peripheral blood vessel blood filling, and analyzed the intestinal flora and metabolism.

**Results:**

Histological analysis showed that the aortic plaque area was significantly reduced in the treatment group. We also found a significant decrease in macrophage accumulation and apoptosis, an increase in expression of scavenger receptors CD36 and SRA1, a decrease in uptake of modified low–density protein (ox-LDL), and a decrease in levels of pro-inflammatory cytokines interleukin (IL)-6 and tumor necrosis factor (TNF)-α following UCSCs treatment. We also found that anti-inflammatory cytokines IL-10 and transforming growth factor (TGF)-β expression increased in the aorta atherosclerotic plaque of the treatment group. UCSCs treatment improved the early peripheral blood filling, reduced the serum lipid level, and inhibited inflammation progression by regulating the intestinal flora dysbiosis caused by the high-fat diet. More specifically, levels of the microbiota-dependent metabolite trimethylamine-*N*-oxide (TMAO) were down-regulated in the treatment group.

**Conclusions:**

UCSCs treatment alleviated atherosclerotic plaque burden by reducing inflammation, regulating the intestinal flora and TMAO levels, and repairing the damaged endothelium.

## Background

Atherosclerosis (AS) is a systemic disease that is associated with the accumulation of lipid-laden macrophages in the arterial wall, which leads to arterial wall thickening and lumen narrowing, subsequently resulting in heart disease, cerebral infarction, and other severe complications [1]. AS is considered a chronic inflammatory disease [2, 3] because an inflammatory response is initiated by the damaged endothelium followed by increased inflammatory cell activation [4]. Macrophages play a critical role in the pathophysiology of AS because they phagocytose cholesterol lipoproteins, forming foam cells that produce cytokines [1]. Macrophages can polarize into two distinct functional phenotypes: M1 and M2 [5]. M1 polarization supports the production of pro-inflammatory cytokines, such as tumor necrosis factor (TNF)-α and interleukin (IL)-6 [5], while M2 polarization leads to secretion of anti-inflammatory factors, such as IL-10 and transforming growth factor (TGF)-β [1, 5].

There is overwhelming evidence that gut microbes and their metabolites contribute to AS pathogenesis [6]. Lipid levels are associated with gut microbiota composition, and dietary composition is central to the metabolic output of the gut microbiota [7]. Thus, if the microbiota is perturbed by environmental or dietary stresses (referred to as dysbiosis), it can increase inflammation and alter metabolism in the host [8]. Microbiota and AS are linked by the production of trimethylamine-N-oxide (TMAO), the oxidized form of trimethylamine (TMA) (a microbiota-dependent detrimental metabolite derived from diets). TMAO is synthesized in the liver, and it has been shown to enhance atherosclerotic lesions by increasing the expression of scavenger receptors, such as CD36 and SRA1, on macrophages [9]. These receptors have high sensitivity to modified lipoproteins and can induce the uptake of modified low–density lipoprotein (ox-LDL), reducing cholesterol efflux in macrophages resulting in foam cell formation [10, 11].

Mesenchymal stem cells (MSCs) have been demonstrated to be effective in the treatment of various diseases, including AS, due to their tissue repair, anti-inflammatory, and immunological properties [12]. MSCs can be isolated from different tissues, including bone marrow, umbilical cord, placenta, adipose tissue, and human gingiva [13–16]. MSCs used for the treatment of atherosclerotic plaques are mostly derived from the bone marrow [17], but also from the gingiva [18], skin [19], cord blood [20], and amnion [12]. Previous studies suggested that MSCs can regulate various inflammatory cells, including macrophages, and inhibit plaque formation by inhibiting inflammatory responses. Human umbilical cord MSCs, referred to as umbilical cord stem cells (UCSCs) in this study, are multipotent cells with a high capacity to differentiate and proliferate [21]. UCSCs can differentiate into cardiomyocytes, skeletal muscle cells, endotheliocytes, and neurons and have been applied in studies of osteochondral, musculoskeletal, and bone tissue regeneration [22–25]. Unlike many stem cell populations, there are few limitations for isolating UCSCs [26, 27]. Specifically, since human umbilical cords are often discarded after childbirth and there are no invasive procedures needed to collect umbilical cord samples, there are fewer ethical issues around sample acquisition [28]. It is also important to note that xenotransplantation of allogeneic UCSCs has been shown to be safe in the treatment of several diseases [29, 30].

To the best of our knowledge, no study has reported the use of UCSCs in the treatment of AS. Therefore, we intravenously transplanted UCSCs in a high-fat diet rabbit model to observe their therapeutic effect and understand their mechanism of action. We found that UCSCs transplantation reduced inflammation in the aortic atherosclerotic plaques by regulating the production and polarization of macrophages, repairing damaged endothelial cells (EC), correcting the intestinal flora imbalance, and preventing harmful metabolite production caused by the high-fat diet. The findings presented here offer new therapeutic insights into to use of UCSCs for treating AS.

## Methods

### Rabbits

Japanese big-ear white rabbits (4–5 months old; weighing 2.3–2.5 kg; 1:1 male to female) were purchased from Beijing Fu Long Teng Fei Experimental Animal Research Institute Co., Ltd. (SCXK [Jing] 2018-0009). Rabbits were randomly divided into three groups: normal, model, and treatment; each group included 5 females and 5 males. The animals were housed in separate cages in ordinary animal house facilities (SYXK [Jing] 2017-0027) and had free access to food and water. The use of animals was in compliance with the Health Guide of Animal Welfare of the National Institutes for Animal Care and Use, and the experiments were approved by the Institutional Animal Care and Use Committee (IACUC) of the Institute of Laboratory Animal Science (permit number: QC19020). All procedures were conducted according to institutional guidelines.

### Cells and culture

UCSCs were obtained from Cyagen Biosciences Inc. (HUXUC-01001, Cyagen). The UCSCs were placed in a 6-well plate and cultured [31] in MEM (12571071, GIBCO) complete medium containing 10% fetal bovine serum (FBS; 10099141C, GIBCO) and 1% penicillin-streptomycin (15140122, GIBCO) with 5% CO_2_ at 37 °C. The media was changed every three days until the cells were confluent, and the cells were then digested and passaged using pancreatin (25200056, GIBCO).

Cells at passage 3 (P3) were collected to identify UCSCs surface markers including CD90-fitc (Invitrogen, Article No. 11-0909-41), CD29-pe-cy5 (BD PHOSFLOW, Article No. 559882), CD73-pe (Invitrogen, Article No. 12-0739-41), CD105-apc (Invitrogen, Article No. 17-1057-41), and HLA-apc-cy7 (Invitrogen, Article No. 47-9956-41). Cells were incubated in 100 μL staining solution at 4 °C for 30 min. We added 1 ml phosphate buffered saline (PBS) and centrifuged the cells at 400×*g* for 5 min. The supernatant was discarded, and the cells were suspended in 200 μL PBS for flow cytometry (FCM) analysis (Becton Dickinson, Aria II).

### Animal model of AS and treatment regimens

Rabbits in the model and treatment groups were fed a high-fat diet containing 0.5% cholesterol, 5% lard oil, 5% yolk powder, 0.2% pig bile salts, and 89.3% basal feed (Beijing Keaoxieli feed Co,. Ltd), 150 g per day for 16 weeks. After 1 month of high-fat diet, rabbits in the treatment group were intravenously injected with UCSCs via the ear vein at a dose of 6 × 10^6^ in 500 μL PBS, once every 2 weeks for 3 months [17, 32]. Rabbits in the model group were given the same amount of PBS under the same conditions. Rabbits in the control group were provided general maintenance and received no treatment or PBS.

### Serum biochemical detection

At 1, 2, and 3 months after treatment, blood samples were collected in normal serum tubes (BD Vacutainer, USA). We used enzymatic detection methods to assay serum triglyceride (TG), total cholesterol (TC), and low-density lipoprotein cholesterol (LDL-C) levels, the alanine substrate method to measure alanine transaminase (ALT), antibody inhibition methods to measure creatine kinase MB (CK-MB), and immunity transmission turbidity with an automatic biochemical analyzer to measure apolipoprotein B (ApoB) (Beckman AU5800, USA).

### Analysis of Doppler flow imaging of peripheral vessels

At 1, 2, 3, and 4 months following high-fat diet, rabbit ears were shaved to remove hair and the blood vessels were photographed. Blood flow in the peripheral vessels was analyzed using laser Doppler flow imaging at 2 months after high-fat diet (Moor FLPI-2, England).

### Vascular ultrasonic evaluation

The aortic arch, carotid artery, and abdominal aorta in each rabbit were imaged using a color Doppler ultrasound diagnostic system (SIEMENS, ACUSON SC2000) 3 months after high-fat diet.

### Histopathological analysis

After treatment, animals were anesthetized and euthanized as previously described [33]. The entire aorta was dissected from the descending aorta to the bifurcation of the common iliac arteries and then fixed with 10% formalin. Adventitial fat and extraneous tissue were dissected. The aorta was split open longitudinally and stained with oil red O staining method (O0625, Sigma) [34]. The percentage of aortic atherosclerotic plaque area (oil red O-positive lesion areas/the planimetry of the entire aortic surface area × 100%) was quantified using Image J 1.42 (NIH, USA). Three sections from each artery were clipped for pathological analysis, and three visual fields were randomly selected for image analysis. Hematoxylin-eosin (HE) staining was used for histopathologic analysis [35]. Immunohistochemistry staining [33] of the aortas included UCSCs antigen stem 121 (Y40410, Takara), ox-LDL (TS2004R, Yaji Biological Co., Ltd.), scavenger receptors CD36 (bs-8873R, Bioss) SRA1 (bs-6763R, Bioss), the EC marker CD31 (ab199012, abcam), the proliferating cell associated antigen Ki67 (ab15580, abcam), the macrophage marker CD68 (MD11047, MDL), inflammatory molecules IL-6 (bs-6312R, Bioss) and TNF-α (bs-2150R, Bioss), and anti-inflammatory molecules IL-10 (bs-0698R, Bioss) and TGF-β (bs-4538R, Bioss). Terminal deoxynucleotidyl transferase (TDT)-mediated dUTP nick end-labeling (TUNEL) was used to detect apoptotic cells using the ApopTag®Plus Peroxidase InSitu Apoptosis Detection Kit (S7101, USA & Canada). All experimental procedures were carried out according to the manufacturers’ instructions. Sections were observed using a light microscope (BX51, Olympus).

### 16S rDNA microbial community analysis

Before the animals were euthanized, fresh feces were taken from each group for intestinal 16S rDNA microbial community analysis. This detection was carried out by Beijing Ouyisaisi Biotechnology Co., Ltd. Diversity analysis of alpha and beta in the intestinal flora and the comparison of relative abundance of the TOP15 flora at the genus level were analyzed.

### TMAO targeted metabolism detection

Animals in each group were euthanized, and a small piece of fresh liver tissue (about 0.5 cm^3^ and 0.03 g) was collected. TMAO metabolite detection was carried out using liquid chromatography tandem mass spectrometry (LC-MS/MS) (Shanghai Luming Biotechnology Co., Ltd). The procedure was done as follows: sample grinding (JXFSTPRP-24/32), purification (SB-5200DT), enrichment, purification, Ultra Performance Liquid Chromatography (UPLC) separation (AB ExionLC), MS/MS Multi-response mode (MRM) detection (AB Sciex Qtrap 6500+), data collection, and qualitative and quantitative analyses of metabolites.

### Statistical analysis

Quantitative analyses for histological studies were performed using Image-pro-plus software (Media cybernetics, Inc. IPP 6.0, USA) and Image J 1.42 (NIH, USA). Statistical analyses were performed using SPSS 16.0 software (SPSS Inc., Chicago, IL, USA) and Microsoft Excel (Microsoft Corporation, Redmond, WA). Continuous data are presented as mean ± standard deviation (SD). A single-factor one-way analysis of variance (ANOVA) was used to evaluate inter-group differences. Differences were considered significant when the *P* value was < 0.05.

## Results

### Characterization of UCSCs

**Fig. 1:**
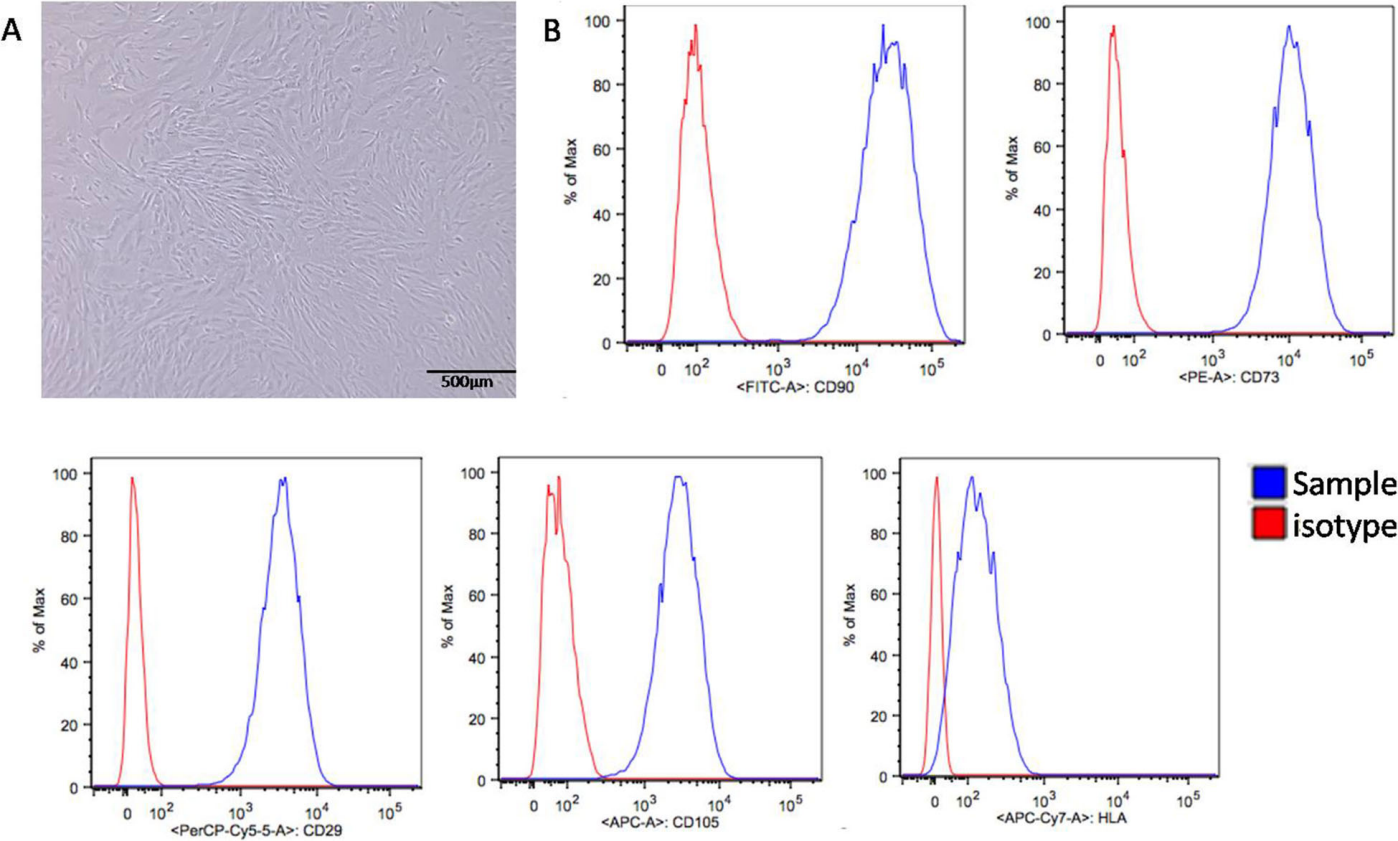
Characterization of UCSCs. **A** UCSCs display a spindle shaped and fibroblast-like morphology. **B** High UCSCs expression of CD90, CD29, CD73, and CD105, and low expression of HLA using flow cytometry

UCSCs at P3 were collected for morphological observation. Phase contrast microscopy showed that the UCSCs had a spindle shape and a fibroblast-like morphology (Fig. 1A). FCM detection of UCSCs surface marker molecules showed high expression of CD90, CD29, CD73, and CD105, and low expression of HLA (Fig. 1B).

### Serum lipid analysis

**Fig. 2:**
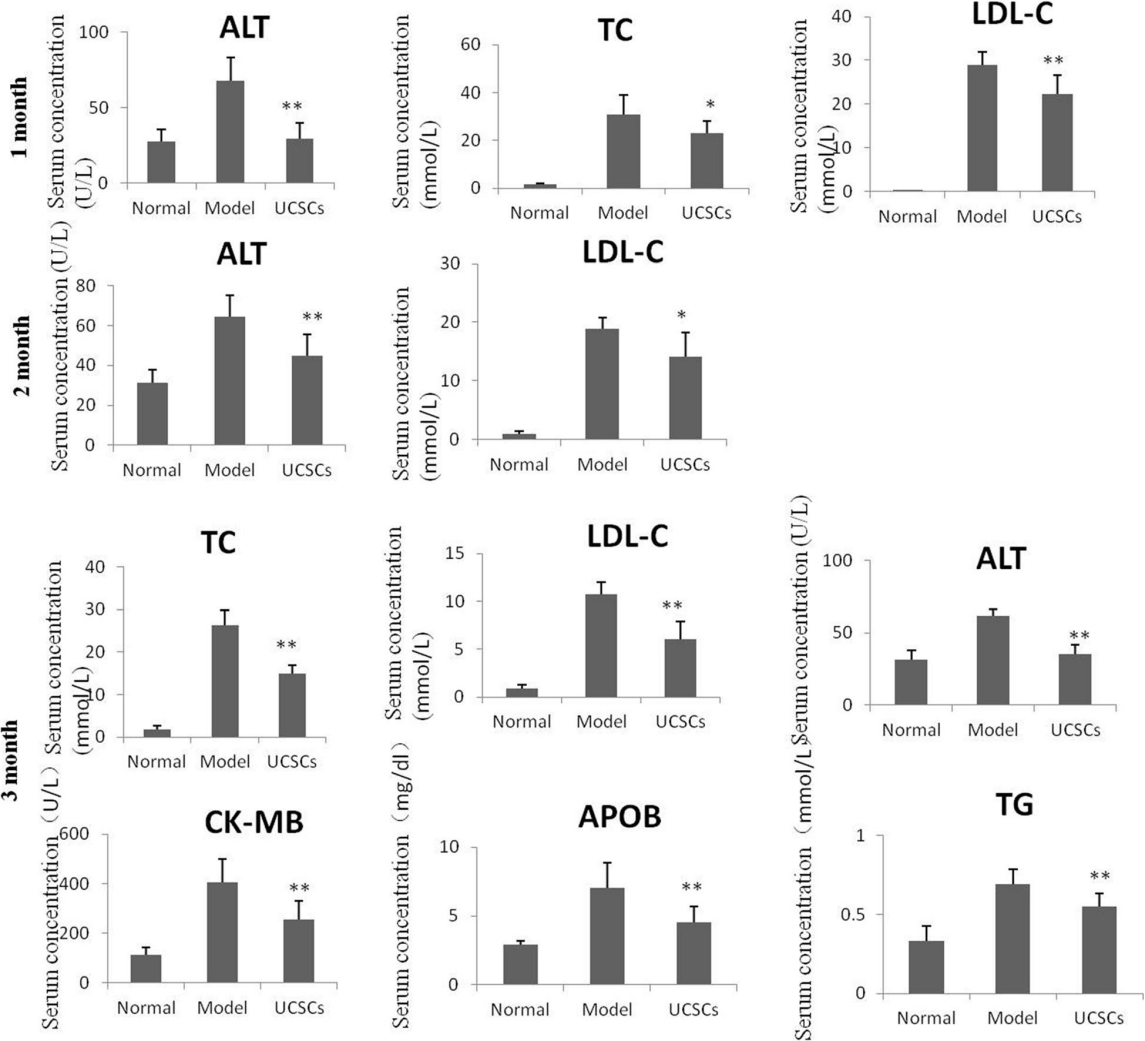
Serum indexes at different time points of UCSCs treatment. Compared with the model group, after 1 month of UCSCs treatment, serum ALT, TC, and LDL-C levels were significantly lower. After 2 months of UCSCs treatment, serum ALT and LDL-C levels declined. After 3 months of UCSCs treatment, serum ALT, TC, LDL-C, TG, CK-MB, and ApoB levels were all significantly lower. (n = 8 in each group) *P < 0.05, **P < 0.01, compared with model group

After 1 month of UCSCs treatment, the serum ALT (*P* < 0.01), TC (*P* < 0.05), and LDL-C (*P* < 0.01) levels in the treatment group were significantly lower than those in the model group. At 2 months after UCSCs treatment, serum ALT (*P* < 0.01) and LDL-C (*P* < 0.05) levels declined in the treatment group compared to those in the model group. After 3 months of UCSCs treatment, serum ALT (*P* < 0.01), TC (*P* < 0.01), LDL-C (*P* < 0.01), TG (*P* < 0.01), CK-MB (*P* < 0.01), and ApoB (*P* < 0.01) levels in the treatment group were all significantly lower than those in the model group (Fig. 2).

### Analysis of Doppler flow imaging of peripheral vessels

**Fig. 3:**
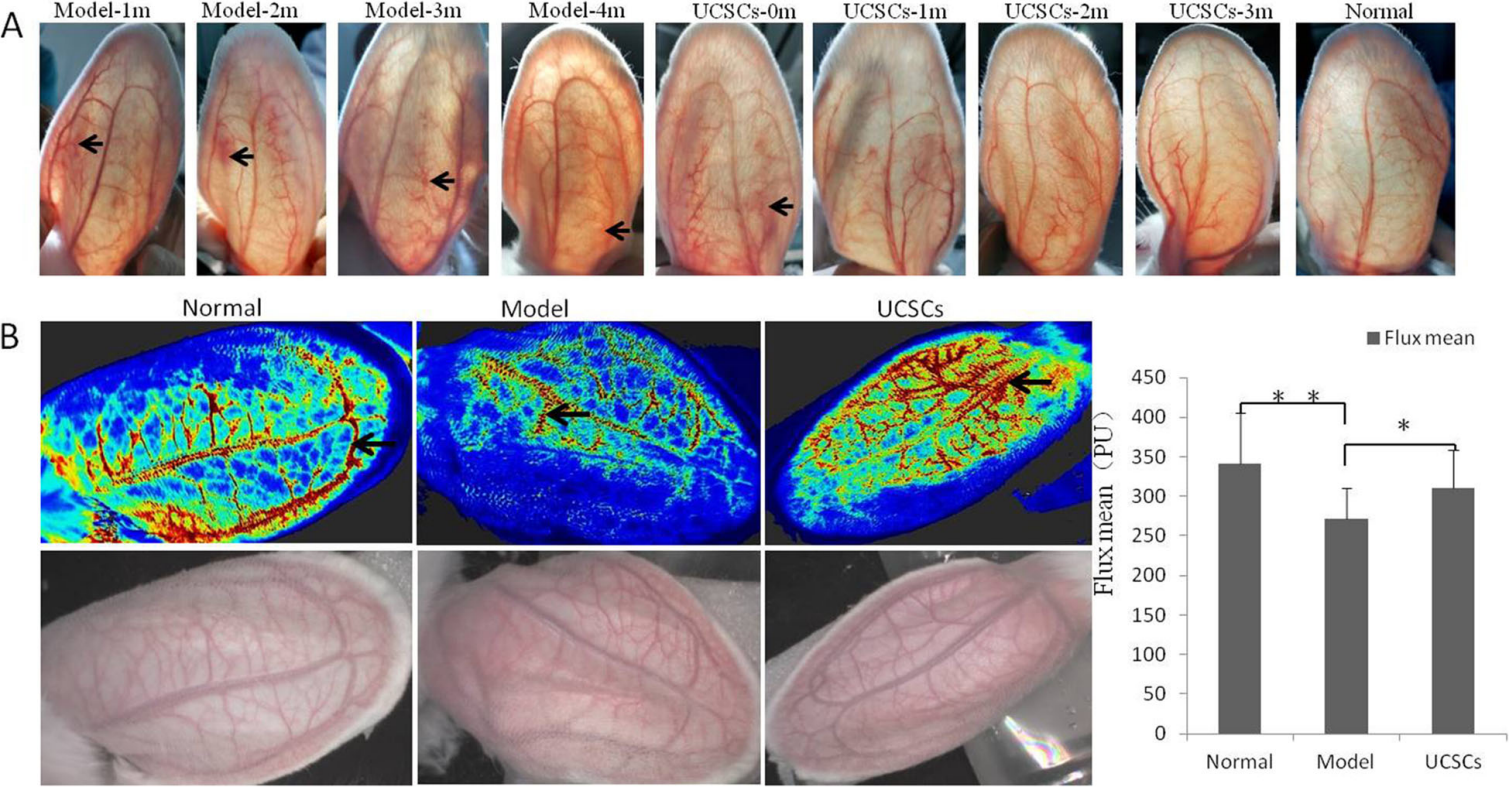
Detection of blood filling of peripheral blood vessels in rabbit ears. **A** Morphological changes of ear peripheral blood vessels at different time points. The blood flow of the model group was not unobstructed (shown by the arrow) after 1, 2, 3, and 4 months of high-fat diet. **B** Analysis of laser Doppler flow imaging. Intravascular hemoperfusion (black arrow) in the model group was less than that of the control and UCSCs groups, and the average blood flow filling value (flux mean) was significantly reduced compared with the control and treatment groups. (n = 10 in each group) *P < 0.05, **P < 0.01, compared with model group

Imaging of the blood vessels in the three groups at the different time points demonstrated that the blood flow in the model group was obstructed after 1 month of high-fat diet, and there were still different degrees of impaired blood flow after 2, 3, and 4 months of high-fat diet. These obstructions were reduced in the treatment group (Fig. 3A).

We further verified blood filling in the rabbit ears after  2 months of high-fat diet. Laser Doppler flow imaging analysis showed that the ear blood filling in the model group was lower compared to the control group, and the average blood flow filling value (flux mean) was significantly reduced (*P* < 0.01). Correspondingly, the average blood flow filling value of the treatment group was higher compared to the model group (*P* < 0.05) (Fig. 3B).

### Vascular ultrasonic detection of atherosclerotic plaques

**Fig. 4:**
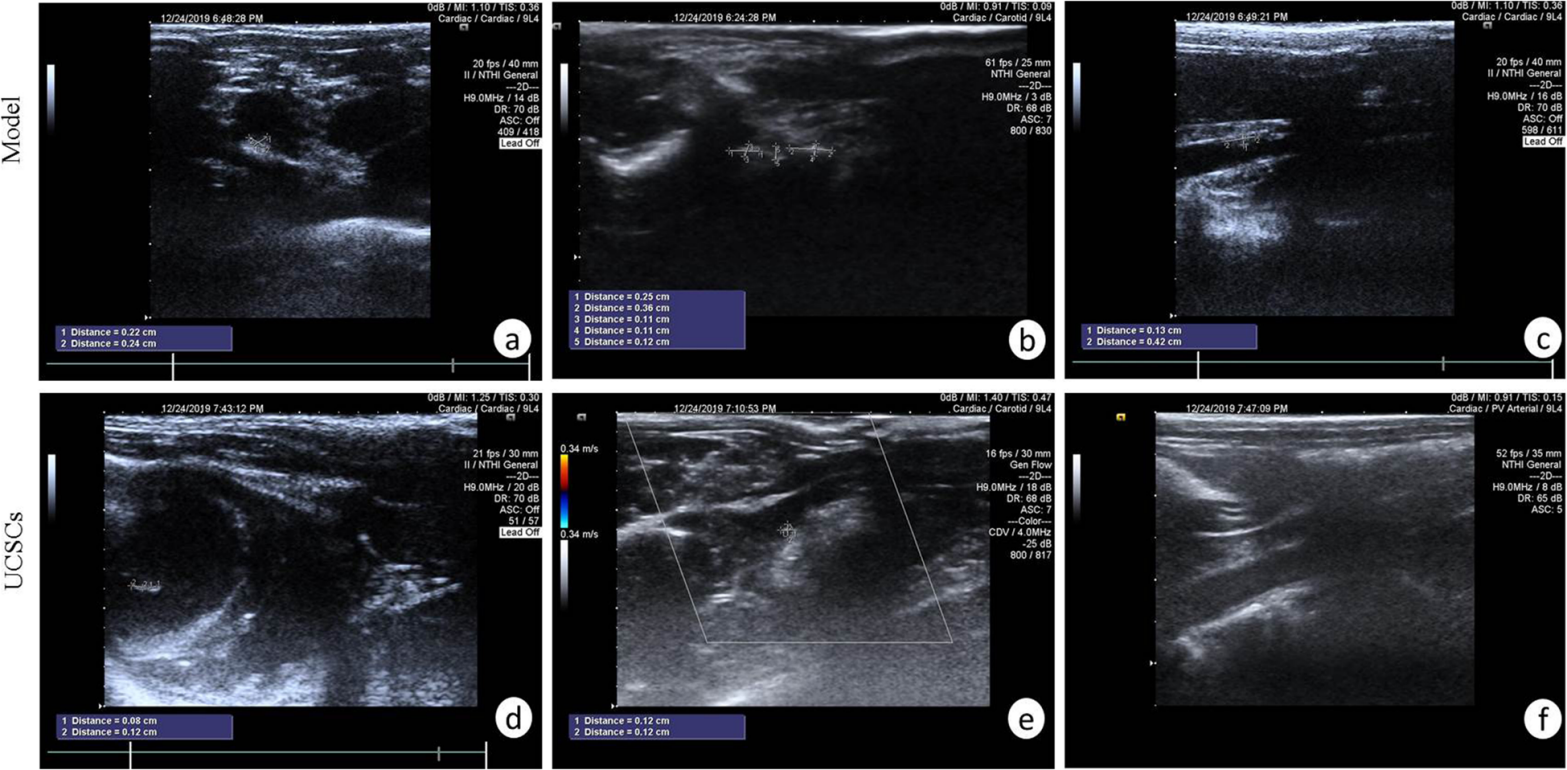
Ultrasonic detection of abdominal aorta and carotid artery. **A** Plaque in the aortic arch. **B** Several small plaques at the carotid artery bifurcation. **C** Plaque in the middle part of the abdominal aorta. **D** Hyperechoic plaque of aortic valve leaflet. **E** Small plaques at the carotid artery bifurcation. **F** Normal abdominal aorta (n = 10 in each group)

After 1 and 3 months of high-fat diet, we performed vascular ultrasonic analysis. After 1 month of high-fat diet, we did not detect any atherosclerotic plaques in the aorta, carotid artery, or abdominal aorta in the model group and UCSCs treatment group. After 3 months of high-fat diet, the aortic arch, carotid artery, and abdominal aorta in the model group showed different degrees of atherosclerotic plaque burden, with incidences of 80% (8/10), 60% (6/10), and 70% (7/10), respectively. Atherosclerotic plaques were also observed in the treatment group, with incidences of atherosclerotic plaque in the aortic arch, carotid artery, and abdominal aorta of 40% (4/10), 10% (1/10), and 20% (2/10), respectively (Fig. 4).

### Pathological changes of aortic atherosclerotic plaques

**Fig. 5:**
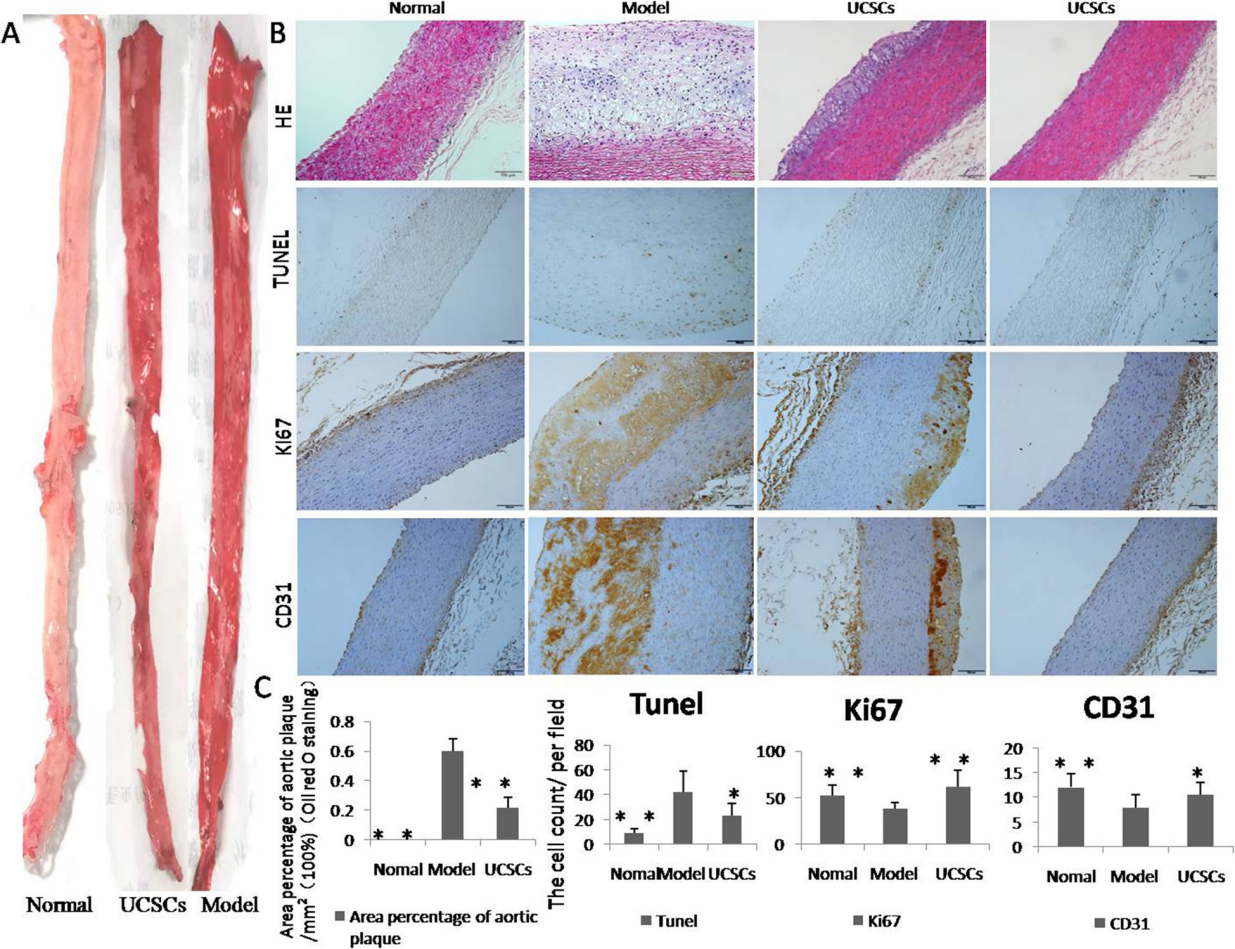
Oil Red O Staining and detection of apoptosis and proliferation in the aorta. **A** Oil red O staining of the aorta; the percentage area of aorta lipid plaque in the model group was significantly larger than that in UCSCs and normal group. **B** HE results show there was different degree of lipid deposit and thickened intima wall in the model group. Apoptosis (TUNEL) of arterial cells decreased, proliferation (ki67) increased, and aortic EC antigen (CD31) expression increased in the UCSCs group. The last column is a representative display showing that most areas of the aorta did not produce plaques in the treatment group, bar = 100 μm. n = 8 in each group. *P < 0.05, **P < 0.01, compared with model group

**Fig. 6:**
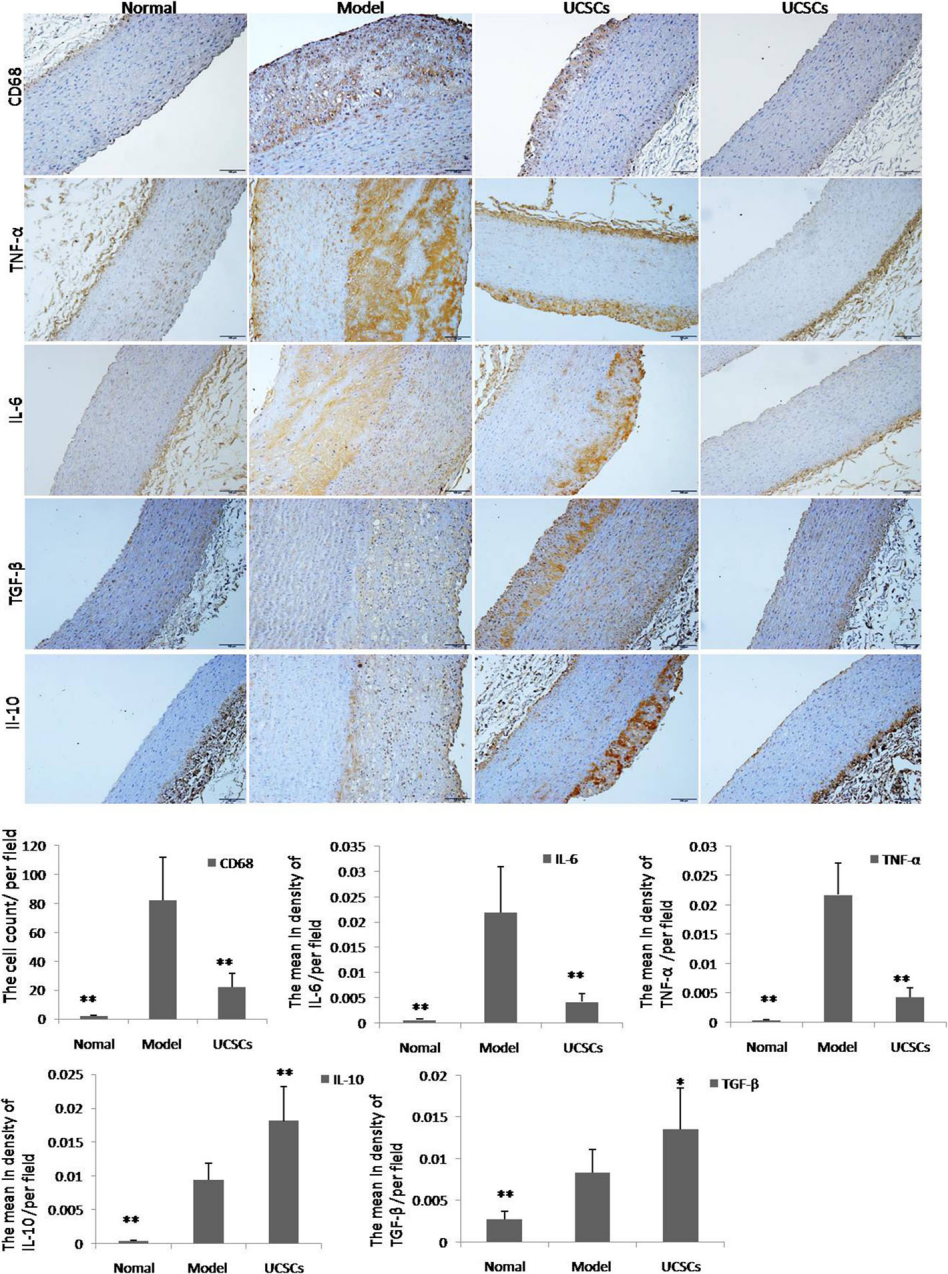
Detection of inflammatory cells and cytokines in aorta. Staining of macrophages (CD68), inflammatory factors (TNF-α and IL-6), and anti-inflammatory factors (TGF-β and IL-10) in the aorta. The last column of Fig. 3 is a representative display showing that most areas of the aorta did not produce plaques in the treatment group, bar = 100 μm. Expression levels of CD68, TNF-α, and IL-6 were significantly decreased, and IL-10 and TGF-β levels were significantly increased in the UCSCs group compared to the model group. n = 8 in each group. *P < 0.05, **P < 0.01, compared with model group

The percentage area of aorta lipid plaque in the model group was significantly larger than that in the UCSCs treatment group, as evidenced by oil-red O staining (*P* < 0.01) (Fig. 5A). Pathological analyses of aortic plaque slices revealed that there were different degrees of lipid deposits, thickening of the intima wall, and greater inflammatory cell infiltration in the aortas of the model group compared to the normal and UCSCs treatment groups. We also found that apoptosis of arterial cells decreased (*P* < 0.05), cell proliferation increased (*P* < 0.01), and EC marker expression increased (*P* < 0.05) in the UCSCs treatment group (Fig. 5B, C). Our inflammatory cell and cytokine detection results showed that levels of the macrophage marker CD68 (*P* < 0.01) and the inflammatory factors IL-6 (*P* < 0.01) and TNF-α (*P* < 0.01) were significantly increased in the model group compared to the UCSCs treatment group. Immunosuppressive factors IL-10 (*P* < 0.01) and TGF-β (*P* < 0.05) were significantly decreased in the model group compared to the UCSCs treatment group (Fig. 6).

### Changes in intestinal flora

**Fig. 7:**
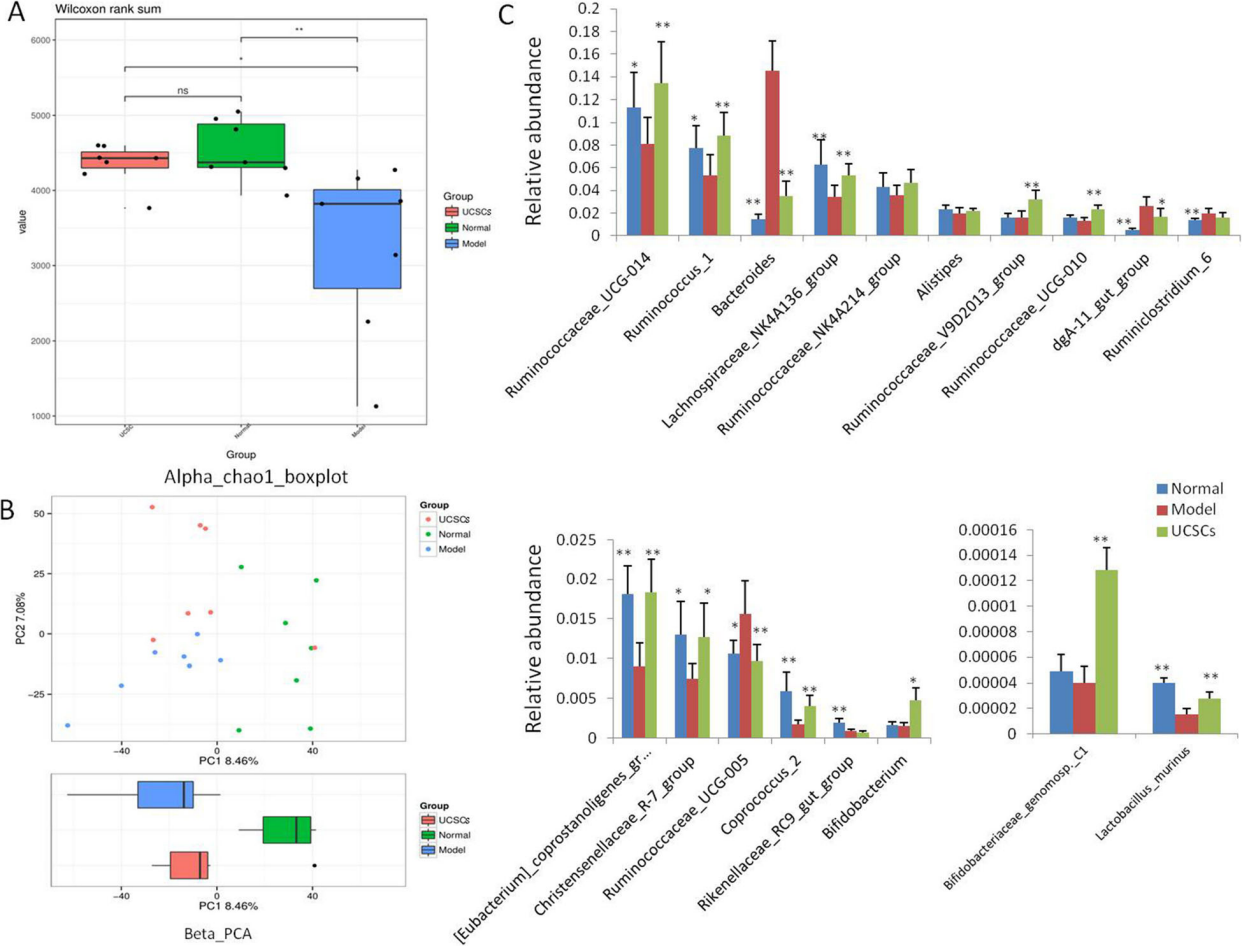
Intestinal flora analysis. **A** Alpha diversity analysis of intestinal flora in each group of animals. **B** Beta-PCA diversity analysis of intestinal flora in each group. **C** Comparative analysis of the relative abundance of the TOP15 intestinal flora in each group. n = 7 in each group. *P < 0.05, **P < 0.01, compared with model group

The results of our TOP15 flora analysis at the genus level showed that there were more abundant microbial colonies in the treatment group. We also found that the diversity index had some differences (Fig. 7A). We found that UCSCs can regulate the imbalance of intestinal flora of rabbits caused by high-fat diet to some extent. In the analysis of relative abundance of multiple bacterial genuses, we found that the relative abundance of 11 bacterial genuses in the UCSCs treatment and normal groups were significantly different compared to the model group (*P* < 0.05 or *P* < 0.01), while there was no obvious difference between the UCSCs treatment and normal groups (Fig. 7B).

### TMAO targeted metabolic analysis and stem cell, ox-LDL, and scavenger receptor detection

**Fig. 8:**
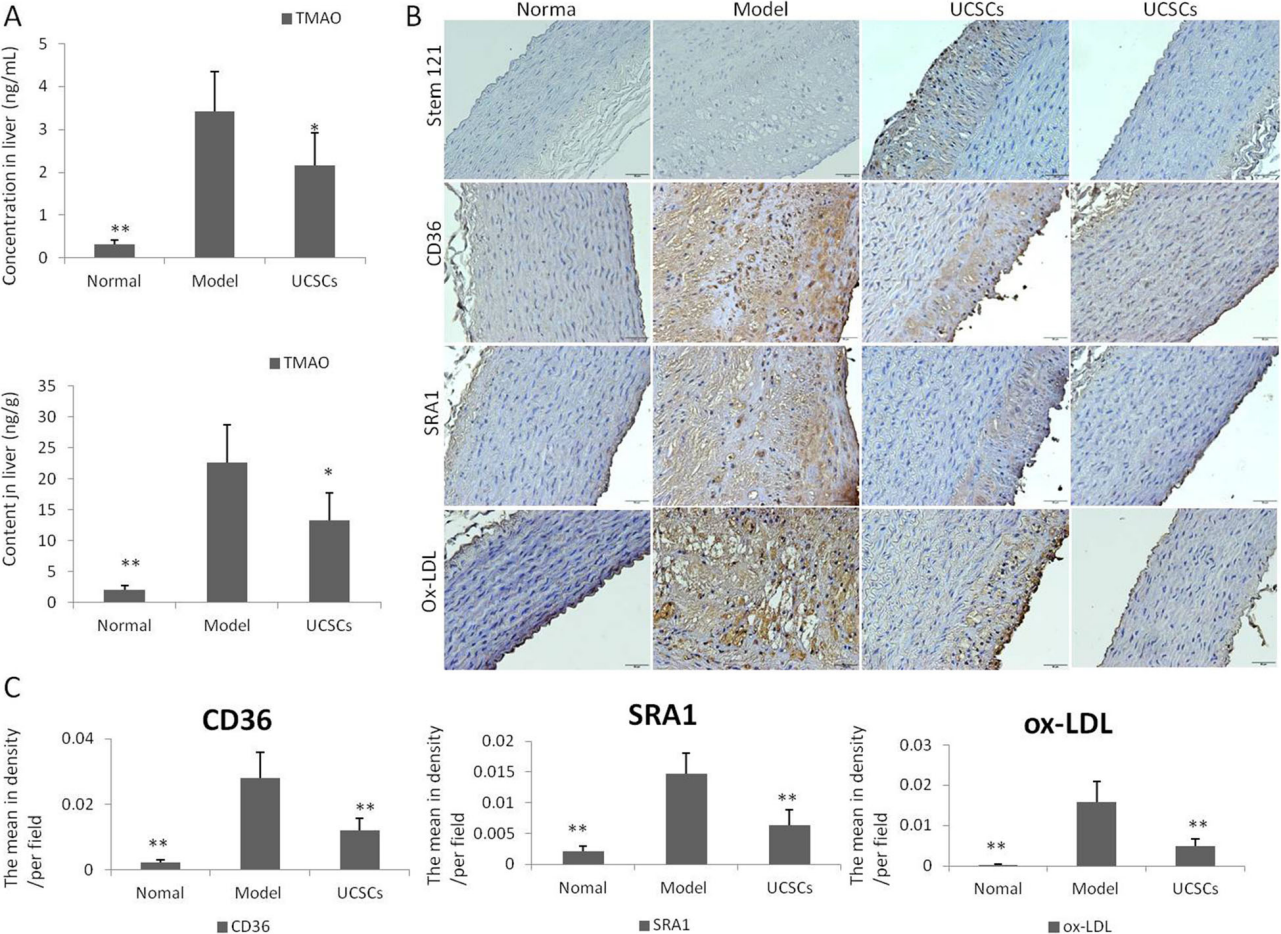
Detection of TMAO, stem cells, ox-LDL, and scavenger receptor in aorta. **A** Quantitative analysis of TMAO secretion in the liver indicated that the concentration (*P* < 0.05) and content (*P* < 0.05) of TMAO in the UCSCs group were lower than that in the model group. **B**, **C** Location analysis of stem cells in the aorta showed that they were mainly located in the aortic plaque of the arterial wall. In the UCSCs treatment group, the absorption of ox-LDL decreased (*P* < 0.01), and the expression levels of macrophage scavenger receptors CD36 (*P* < 0.01) and SRA-1 (*P* < 0.01) decreased in the aorta. n = 6 in each group.*P < 0.05, **P < 0.01, compared with model group

Quantitative determination of the metabolite TMAO in the liver tissues using UPLC-MS/MS indicated that the concentration (*P* < 0.05) and content (*P* < 0.05) of TMAO in the UCSCs treatment group were significantly lower compared to the model group (Fig. 8A).

The location analysis of stem cells in the aorta showed that the cells were mainly located in the aortic plaque of the arterial wall (Fig. 8B). In the UCSCs treatment group, there were significant decreases in the absorption of ox-LDL (*P* < 0.01) and the expression of macrophage scavenger receptors CD36 (*P* < 0.01) and SRA-1 (*P* < 0.01) (Fig. 8B, C).

## Discussion

The use of MSCs has increased in the clinical treatment of various diseases. However, for an MSC population to be effective clinically, it must have low antigenicity [36]. Since autologous and allogeneic UCSCs have been shown to be safe in the treatment of many diseases [22–25], we investigated if UCSCs could be used to effectively treat AS in this study. We found that UCSCs treatment can decrease inflammation by regulating the gut flora and reducing harmful metabolite production in a rabbit model of AS. Thus, our study indicates that UCSCs treatment may be a promising strategy to attenuate atherosclerotic lesion development in humans.

In this study, we used an in vivo high-fat diet model to induce AS in rabbits. Cholesterol-fed rabbits are widely used for AS studies [37] because of their unique characteristics, including sensitivity to dietary cholesterol, their similarities with human lipoprotein metabolism [37], and the fact that the high-fat diet better simulates the natural process of AS in humans.

Cholesterol, TG, and lipoproteins are known factors in atherosclerosis pathogenesis. Increased serum LDL and TG concentrations are responsible for the formation of atherosclerotic lesions [38]. APOB is a kind of plasma lipoprotein that can carry chylomicrons, which are very low-density lipoproteins (VLDL), and LDL into blood vessels. The accumulation of LDL and VLDL in blood vessels is increased following increases in plasma APOB [39]. In cases of hypercholesterolemia, monocyte-derived macrophages infiltrate the arterial intima to clear the APOB containing lipoproteins (e.g., LDL) and are transformed into lipid-laden macrophage foam cells [40]. Here, we transplanted UCSCs 4 weeks after the start of the high-fat diet, and the effect of early intervention on AS diseases was observed. We found that serum TC and LDL-C levels decreased after 1 month of UCSCs transplantation and serum TC, TG, and LDL-C levels decreased after 3 months of treatment. ALT, APOB, and CK-MB levels were also lower in the treatment groups compared to the model group. These data suggest that UCSCs transplantation has the potential to attenuate atherosclerosis by reducing serum TC, TG, and other lipid levels.

It has been reported that gut microbes and their composition can regulate lipid metabolism (including fatty acids, TG, and cholesterol) [7, 41]. A decrease in total microbial abundance in the intestine is associated with increased serum TC and TG levels [42]. *Bifidobacterium* spp. can reduce circulating TG and LDL levels and increase HDL levels [43]. Experiments in high-fat diet mice also showed that changes in intestinal flora affect serum lipid levels, and some bacteria in the *Lactobacillus* species can reduce plasma cholesterol levels [44]. TMAO, a microbial-dependent metabolite, can suppress reverse cholesterol transport and increase serum cholesterol levels [45, 46]. In our research, we found that UCSCs can regulate intestinal flora homeostasis, increase the abundance of intestinal flora, increase the levels of *Bifidobacterium* and *Lactobacillus*, and reduce the level of TMAO synthesis. Therefore, our findings indicate that serum lipid levels are reduced to some extent by UCSCs treatment.

Much of the current evidence indicates that macrophages participate in AS pathogenesis [47], as their accumulation in endothelial lesions is an important pathological change in AS [48]. Macrophages form foam cells after taking up LDL in the vascular intima [47]. Macrophages also help maintain the local inflammatory response by secreting pro-inflammatory cytokines and chemokines and producing reactive oxygen species. Dying macrophages are responsible for necrotic core formation in progressing plaques [49]. Therefore, reducing macrophage aggregation and the formation of foam cells in atherosclerotic plaques is essential for controlling the inflammatory response during AS. In this study, we observed atherosclerotic plaque formation in the aortic intima in the model and UCSCs groups. The aortic plaque area percentage, the expression of macrophages, and the apoptotic cell number were significantly decreased in the UCSCs treatment group compared to the model group. It has been reported that MSCs can migrate to injured tissue, interact with injured host cells, and secrete paracrine-soluble and growth factors that modulate immune responses and alter endothelial responses [50]. Here, we found that the number of proliferating ECs in the treatment group was more than that in the model group, which indicates that UCSCs transplantation reduced macrophage aggregation in the arterial plaque, promoted cell proliferation, and repaired endothelial damage to a certain extent. These findings are important because recovery of the endothelium is critical in the early stage of AS; EC deterioration and reduction can greatly influence the subsequent progression of AS [51].

UCSCs have been shown to inhibit the differentiation and maturation of other important inflammatory cells, namely dendritic cells (DCs) [52], by reducing the expression of proinflammatory cytokines TNF-α and IL-6 and increasing the production of anti-inflammatory cytokines TGF-β and IL-10. These effects indirectly suppress T cell proliferation [53]. TNF-α and IL-6 play central roles in the inflammatory response and are recognized as predictive indicators of plaque instability. Both cytokines can direct inflammatory cells to accumulate in atherosclerotic plaques, negatively impacting plaque stability and promoting thrombosis and cell necrosis [54–56]. IL-10 can promote atherosclerotic lesion stability [57] and decrease the synthesis of TNF-α [58]. In atherosclerotic lesions, TGF-β secreted from macrophages plays a role in vascular biology by affecting cell proliferation, differentiation, migration, adhesion, apoptosis, and extracellular matrix production [59]. Its anti-atherosclerotic processes involve reducing inflammatory cell recruitment, platelet adhesion, and macrophage activation [60]. In our study, UCSCs transplantation decreased the pathological inflammatory response by inhibiting cell apoptosis in vulnerable plaques, reducing the number of macrophages and the levels of pro-inflammatory cytokines TNF-α and IL-6, while increasing the anti-inflammatory cytokines IL-10 and TGF-β. It has been reported that M1 polarized macrophages mainly produce TNF-α and IL-6, and M2 polarized macrophage mainly produce TGF-β and IL-10. Thus, UCSCs may inhibit inflammation by regulating macrophage polarization and cytokine secretion.

Emerging data suggest that intestinal flora dysbiosis can contribute to AS development by increasing systemic inflammation [61–63]. A high-fat diet can induce gut flora dysbiosis, which can lead to increased inflammation and altered metabolism in the host [64]. Gut barrier integrity is essential for maintaining the host’s health and preventing inflammation and AS processes [65]. The integrity of the intestinal barrier epithelium can be maintained by the gut microbiome, dietary constituents, and microbiome-derived metabolites, and proinflammatory changes associated with intestinal barrier disruption contribute to metabolic inflammation and immune responses [66]. A high-fat diet can alter the intestinal barrier structure [65] and further increase bacterial translocation [67]. Dysbiosis can increase the intestinal permeability by suppressing tight junction proteins, allowing the translocation of lipopolysaccharide into the circulation [65, 67–70]. Gut dysbiosis derived lipopolysaccharide binds Toll-like receptors (TLRs) and activates downstream immune factors [71]. Upregulation of TLRs initiates the inflammation driven AS process [72, 73], enhancing the synthesis of pro-inflammatory cytokines, such as IL-6 and TNF-α [74, 75]. In addition, the gut microbiota-dependent metabolite TMAO can increase macrophage migration, promote inflammatory cytokine expression, reduce cholesterol efflux, and increase foam cell formation, leading to AS progression [76]. In our study, we found that at the relative abundance of 11 genus intestinal flora from the TOP15 analysis in the model group were in a state of dysbiosis compared to the normal group. In the UCSCs group, the intestinal flora dysbiosis was alleviated to some extent, and TMAO synthesis decreased. These results indicate that UCSCs can antagonize the imbalance of intestinal flora caused by a high-fat diet and down-regulate TMAO levels, thus inhibiting intestinal barrier destruction, inflammatory reactions, and AS progression. Thus, gut permeability and inflammation may be underlying mechanisms by which UCSCs regulate the intestinal flora in AS [77].

The intestinal microbiota and host immune system regulate each other [78]. Therefore, based on the above findings and the literature, UCSCs have the capacity to inhibit inflammation, regulate immune responses, reduce production of TMAO, protect the intestinal barrier, and maintain intestinal flora homeostasis. Ikarashi et al. reported that human adipose and umbilical cord MSCs protected against inflammation and maintained the balance of intestinal flora in the dextran sulfate sodium (DSS)-induced colitis mouse model, which may be related to MSC-derived exosomes [36]. Thus, further studies are needed to identify the specific mechanisms by which UCSCs exert their therapeutic effects in AS.

TMAO generation is dependent on the gut microbiota, and it can exacerbate inflammatory reactions in the vascular wall and impair cholesterol reverse transport [79]. TMAO can increase the prevalence of AS by increasing the expression of scavenger receptors (CD36 and SRA1) and inflammatory cytokines (IL-6 and TNF), as well as increasing macrophage migration [76, 80]. Alterations to the intestinal flora composition can affect TMAO synthesis in the liver [75]. In our experiments, UCSCs inhibited TMAO synthesis through modulating the gut microbiota, but the specific mechanism requires further analysis. The number of macrophages and the levels of TNF-α and IL-6 were reduced in our study, which further indicate that UCSCs can block inflammation by inhibiting TMAO synthesis. The levels of scavenger receptor CD36 and SRA1 and the uptake of ox-LDL by macrophages were also reduced. Therefore, we propose that UCSCs can attenuate atherosclerotic lesion development by maintaining intestinal flora stability, inhibiting the production of intestinal-related harmful metabolites, and preventing ox-LDL phagocytosis.

Previous studies have shown that the formation of atherosclerotic plaques in large-medium size arteries is associated with vascular lesions in peripheral vessels, resulting in reduced blood flow filling or filling obstruction. UCSCs transplantation can improve the morphology and function of peripheral blood vessels in the early stage of AS. Our ultrasound analysis found that the incidence of atherosclerotic plaques in the UCSCs treatment group was lower than that in the model group after 3 months of high fat diet, further supporting the hypothesis that early UCSCs intervention therapy can reduce the formation of atherosclerotic plaques.

## Conclusions

This study demonstrates that UCSCs transplantation in the treatment of AS at the early stages can not only attenuate atherosclerotic plaque formation and progression in large and medium-sized vessels, but also improve early peripheral blood filling. UCSCs transplantation can alleviate AS by reducing the serum lipid level, inhibiting the production of macrophages and inflammatory cytokines (IL-6 and TNF-α), inhibiting apoptosis, promoting production of anti-inflammatory cytokines (IL-10 and TGF-β) and endothelial cells, further inhibiting inflammatory responses and repairing damaged endothelium. UCSCs can also inhibit inflammation progression and ox-LDL phagocytosis by balancing intestinal flora dysbiosis caused by high-fat diet, as well as reducing TMAO production, which can also lessen atherosclerotic plaque burden. Future work will provide and in-depth investigation of the molecular mechanism by which UCSCs regulate intestinal flora and metabolite production to reduce AS progression.

## Data Availability

The datasets used and/or analyzed in the current study are available upon request.

## Abbreviations

AS: Atherosclerosis
MSCs: Mesenchymal stem cells
UCSCs: Umbilical cords mesenchymal stem cells
IL: Interleukin
TNF: Tumor necrosis factor
TGF: Transforming growth factor
TG: Triglyceride
TC: Total cholesterol
LDL-C: Low-density lipoprotein cholesterol
ALT: Alanine transaminase
CK: Creatine kinase
ApoB: Apolipoprotein B
EC: Endothelial cells
